# Silent witnesses: the experience of having a sibling with anorexia nervosa

**DOI:** 10.1186/s40337-022-00655-1

**Published:** 2022-09-06

**Authors:** Suzanne Hutchison, Jennifer House, Beth McDermott, Mima Simic, Julian Baudinet, Ivan Eisler

**Affiliations:** 1 Institute of Psychiatry, Psychology and Neuroscience (IoPPN), King’s College London, 16 De Crespigny Park, London, SE5 8AF UK; 2 Maudsley Centre for Child and Adolescent Eating Disorders (MCCAED), Maudsley Hospital, De Crespigny Park, Denmark Hill, London, SE5 8AZ UK

**Keywords:** Sibling, Anorexia nervosa, Family therapy, Maudsley family therapy, Family based treatment

## Abstract

**Background:**

This study explored the experience of having a sibling with anorexia nervosa and the sibling perspectives on service provision.

**Method:**

Four focus groups were conducted with 14 siblings (8 female, 6 male, age 11–19 years) of adolescents with anorexia nervosa or related restrictive eating disorders. Group discussions were transcribed and analysed using thematic analysis.

**Results:**

Four themes and eight sub-themes were generated. These illustrated siblings feel greatly affected by the way the family needs to change to support someone with anorexia nervosa. Feelings of ambivalence and acceptance were also evident. They described silencing their own emotions and needs so as not to trouble others, and distancing themselves from their families in order to cope. Some female (but no male) siblings identified an impact on their own perceptions of eating and body image. Siblings generally felt that services had not attended to their needs, and that they had not been appropriately included in treatment.

**Conclusions:**

Data from this study suggest the sibling experience needs to be more carefully considered and included in treatment. This may include a more explicit invitation to sessions and a more active discussion about their own needs and useful involvement in treatment sessions. Findings point to ways siblings may be better supported, such as peer support groups.

**Supplementary Information:**

The online version contains supplementary material available at 10.1186/s40337-022-00655-1.

## Plain English summary

This study investigated the experience of having a sibling with anorexia nervosa, and the sibling perspectives on the treatment they get. Four focus groups were held with siblings (11–19 year-olds) of adolescents with anorexia nervosa. Group discussions were written down word-for-word and analysed using thematic analysis. Four themes and eight sub-themes were identified. These illustrated siblings feel very affected by the way the family needs to change to support someone with anorexia nervosa. Feelings of ambivalence and acceptance were also evident. Siblings said they silenced their own emotions and needs so as not to trouble others, and distanced themselves from their families in order to cope. Siblings generally felt that services had not attended to their needs, and that they had not been appropriately included in treatment. Findings from this study point to ways siblings may be better supported, such as peer support groups.

**Supplementary Information:**

The online version contains supplementary material available at 10.1186/s40337-022-00655-1.

## Introduction

The impact of anorexia nervosa on the adolescents and their parents is well-documented [1–4]. The inclusion of parents is an important, recommended part of treatment [4–6]. Less is known about the experiences of siblings and how best to include them in treatment, if at all.

The limited research into siblings of people with anorexia nervosa suggests the experience and impact is similar to those who have a sibling with chronic or serious physical health problems. Available data suggests they can be positively *and* negatively affected [7–9]. Siblings can develop more compassion and maturity, may report increased closeness and communication, all of which may improve relationships. At the same time, illness management regimes and changes in family functioning can result in less time and support from parents [7, 9]. Regarding well-being, siblings of people with eating disorders have reported higher rates of depression and difficult sibling relationships [10], as well as poorer psychological adjustment compared to their peers, even after their sibling has completed treatment [11].

Parents of adolescents with anorexia nervosa have expressed concern that siblings’ needs are neglected by the family and services [12, 13]. Psychoeducation and inclusion in family therapy treatments is often recommended [14, 15] and has been suggested to be helpful, although some distance from the illness is also recommended [16–18]. One small study also suggested that sibling gender may be important to consider, with female, or same-sex, siblings potentially more affected than male siblings [19]. This fits with evidence that same-sex siblings, particularly sisters, report higher intimacy than other sibling combinations [20], and thus may be more impacted by the illness.

While including siblings in family therapy is suggested, in practice attendance is reportedly low and drops off sharply after treatment commences [21]. Sibling attendance is also not associated with improved end of treatment outcomes [21, 22], making it difficult to properly understand how best to involve and support siblings. Available guidance suggests offering an assessment of a sibling’s own needs may be helpful [23], although specifics beyond that are unclear.

Although existing studies are informative, they are few in number. Male siblings and adolescents have tended to be under-represented [9]. The present study aimed to add to this emerging literature by exploring the male and female adolescent sibling perspectives, to understand how they are affected by the eating disorder, and explore their experiences and requirements of services.

## Method

This study was approved by Leeds West NHS Research Ethics Committee (REC: 11/YH/0047). All participants gave informed consent.

### Design

As the topic of interest was participants’ perspectives and experiences, focus groups were used and data were analysed using thematic analysis from a realist epistemological position [24, 25]. This allowed for in-depth exploration of similarities and differences in experiences.

### Participants

Participants were recruited through the Maudsley Centre for Child and Adolescent Eating Disorders (MCCAED), a large specialist child and adolescent eating disorder service in South London [26]. Inclusion criteria were: (1) aged 10–20 years (relatively similar ages to the sibling in treatment); (2) sibling currently receiving treatment for anorexia nervosa or a similar restrictive eating disorder at time of recruitment; (3) lived at home during sibling’s treatment. All adolescents with the eating disorder received family therapy for anorexia nervosa [14] with or without adjunctive multi-family therapy [27], to which their siblings were invited. All siblings attended at least one treatment session.

### Recruitment

Clinical staff wrote to 77 service users asking permission to contact their sibling/s. Twenty-four consented; 53 declined (without giving reasons) or did not respond. Twenty-nine siblings (> 1 from some families) were then contacted directly with written information about the study. Nineteen (65% of siblings contacted) agreed to take part (those who refused did not give reasons), and 14 (48% of siblings contacted) were able to attend a focus group. They were not compensated for participating. None were known to have an eating disorder, but this was not formally assessed. However, this is unlikely as the recruiting service managed all eating disorder referrals in the catchment area, and all participants were seen at least once in family/multi-family therapy, without concerns being raised. More details can be found in Table 1.

**Table 1:** Demographics

*Participant characteristics*	
Gender	8 Female, 6 male
Self-defined ethnicity	12 White British, 1 Mixed Race, 1 British Asian
Mean age (range)	14.9 (11–19) years
Birth order of participant	6 Older than sibling in treatment 8 younger than sibling in treatment
*Characteristics of the sibling with an eating disorder*	
Gender of sibling in treatment	14 Female, 0 male
Mean age of siblings in treatment (range)	15.4 (13–18) years
Mean duration of siblings’ disorders (range)	2.9 (1.7–4.8) years

### Procedure

Data were collected through focus groups, formed according to participants’ ages (two for 10–16 year-olds, two for 17–20 year-olds). The younger groups each consisted of five siblings (two brothers and three sisters in one, four brothers and one sister in the other), and the older groups consisted of two sisters in each. Each group had two facilitators (any two of the authors SH, JH or a research colleague), at least one of whom had training in qualitative research methods and previous experience of running groups. Group discussions were initiated using a flexible, semi-structured topic guide that included prompt questions (see Additional file 1) and lasted approximately 90 min. Discussions were recorded and transcribed verbatim.

### Data analysis

Data were analysed using reflexive thematic analysis from a realist epistemological position, and themes were identified at a more explicit than interpretative level [24, 25]. Author SH initially immersed herself in the data and noted initial ideas. She then summarised each sentence and created an outline of paraphrased items for each transcript. Summaries were compared to generate initial codes, to organise the data into meaningful groups and create a codebook [28]. Inter-rater reliability was established by the third author independently coding the data. Following consensus, analysis of the codes took place to search for common and overarching themes, sub-themes and exemplary quotes, which were reviewed with author JH. The concept of data saturation was not used during the analysis as it was recognised that different meanings are generated by different researchers and the process is inescapably subjective [29]. As such, the concept was also not relevant as a means of identifying an adequate sample size. Rather, the data are considered in the context of the study participants and researchers’ experiences.

## Results

Four themes and eight sub-themes were identified, which are presented in Table 2. Related quotes are provided below, which are written verbatim, with only minor adjustments to spelling to aid ease of reading.

**Table 2:** Themes and sub-themes

Main themes	Sub-themes
1. Changes as a result of anorexia nervosa	1a. Changes in family members 1b. Changes in family life 1c. Changes in relationships
2. Siblings’ role following the development of anorexia nervosa	2a. Helping 2b. Not troubling others 2c. Coping
3. ‘Their needs above yours’	3a. In the family 3b. In services
4. Support for siblings from siblings	

### Theme 1: Changes as a result of anorexia nervosa

Siblings described changes in family members, family life, and relationships as a result of their sisters’ illnesses. In some cases, the impact of the changes as a result of anorexia nervosa were described as having an all-encompassing effect.

#### 1a. Changes in family members

Participants described changes in the behaviour, thoughts and feelings of their ill sisters and parents, and of the participants themselves. Siblings described both a positive and negative impact on themselves, but a predominantly negative impact on others in the family. Siblings described mood swings and changes in their sisters’ character as a result of anorexia nervosa, and exhaustion in their parents, as they attempted to fight their child’s illness.

*“I’m much more sympathetic … I think it’s just made me a better person.” (younger sister)*

*“[My sister] used to be quite confident and she became quite withdrawn.” (older sister)*

*“[It has put a] strain on my parents and they do just generally seem a lot more wearied … my mum just looks really tired, and like everything is really difficult … [my dad] will just snap and he can’t deal with it” (older sister)*

Some female siblings specifically reported a heightened awareness and perceptions of their own eating and body image. Several sisters spoke of feeling watched by their unwell sister when eating or feeling more “guilty” about what they ate. Another sister spoke of valuing a more “womanly, curvy” figure after living in a house with anorexia nervosa. This was not described by any male siblings.

*“I’ve started to think about my weight and the way I look a lot more.” (younger sister)*

*“It’s made me so much aware of what I eat … if you are having a bit of chocolate you can see (sister) over there, looking like how she does and you just, you’re afraid of what she’s thinking, um, but it’s definitely changed the way I eat and how, you know, I feel guilty eating bad things.” (older sister)*

#### 1b. Changes in family life

Participants explained how family life was disrupted, both generally by the change in atmosphere at home, and during more specific family-based activities, such as mealtimes and holidays. Descriptions generally centred on the family’s shared environment. However, a sibling also described how the effect and changes of anorexia nervosa are felt even beyond the family circle.

*“If one person in the family has it then all … are affected by it … before I would use school to take my mind off it … but it started to get so bad that … almost, everything that someone would talk about seemed to relate to it.” (younger brother)*

#### 1c. Changes in relationships

Ambivalence was particularly evident when some participants spoke about their feelings with regards to their relationship with their ill sisters. The group reaction to the mention of such confusion between sadness, care and frustration was one of consensus by means of non-verbal cues such as nodding and shared laughter. All participants spoke of other relationships in the family as having changed as a result of anorexia nervosa by either becoming closer in some cases, or more distant or difficult in others between different family members. No clear pattern of which dyads became closer or more distant was evident.

*[I feel] “resentful … definitely. Very angry. It’s difficult to be angry with anorexia and not [sister].” (older sister)*

*“I’ve definitely grown closer to my sister ‘cause we didn’t really used to get on at all but now I’m like the friend, then my parents are like the people who tell her to eat and stuff so I was like the one who could cheer her up and stuff.” (younger sister)*

### Theme 2: Sibling role

Participants discussed what they felt to be their roles following the development of their sisters’ illnesses. These roles fell into three broad areas: helping their parents with their ill sister, not troubling others, and finding ways to cope with the changes and effects of anorexia nervosa outlined above.

#### 2a. Helping

Siblings all agreed that their parents tried not to put any responsibility for their sister’s well-being on them, but they described efforts to help their parents manage nonetheless. Not being able to help could in some cases lead to siblings feeling guilty, which was particularly present for older siblings. Situations such as a particular behaviour in their sister and additional stressors such as a parent’s illness increased the sense of wanting to support parents as reflected in older siblings’ comments:

*“I almost feel … a little bit guilty [about going to university], because you’re like leaving the family … I think they find it quite useful for me to be around” (older sister)*

*“She has gotten more and more violent with it, so then you kind of feel you have to take some of it, ‘cause like the parents can’t do it all the time … I guess as the older sibling you feel like you kind of have more a thing to protect them and help them” (older sister)*

*“I don’t feel like … it should be my responsibility, but em, my mum’s actually quite ill …I do want to take responsibility so that my mum doesn’t have to.” (older sister)*

#### 2b. Not troubling others

A consistent response from participants was the sense that it was their role to keep their needs or feelings to themselves so as not to trouble or ‘burden’ others. This was reflected in relation to their parents and beyond that, with their own friends.

*“I don’t really talk about it to my friends ‘cause … to explain it all, it just sounds like you’re just moaning on about it.” (older sister)*

*“[I] always have the feeling of not really wanting to put my problems on [parents], ‘cause obviously they’ve been so preoccupied with my sister … if it was just me and [them] … I’d have to hold some things back so that they didn’t feel too guilty.” (younger sister)*

#### 2c. Coping

Participants described coping as part of their role. The coping mechanism employed by most siblings of distancing themselves from the family home follows from the idea of not causing trouble or staying out of the way and ‘getting on with things’. Siblings’ responses reflect how finding a sense of normality by being away from the home was comforting to them.

*“Yeah, it’s kind of like comforting going to friends’ houses because with their siblings they have like petty fights about, like, going into each other’s rooms … I never really had that because it’s so much bigger things … [so] I’ve been like staying out much later and … spending a lot of time away from home recently.” (younger sister)*

### Theme 3: ‘Their needs above yours’

Participants believed that their needs or feelings were treated as secondary by others, such as family members and services. The general reaction to this was one of acceptance of it being necessary.

#### 3a. In the family

*“‘Cause again it’s such a long illness, you’re kind of just like shoved out and you’re like on your own a bit, but because you’re worried about them … you’ve just got to get on with it really.” (younger sister)*

*“Yeah, I think it’s always their needs above yours … not that your parents [are], like, being intentionally, like, neglectful of you, but … I always understood that, that that’s how it needed to be.” (younger sister)*

#### 3b. In services

Responses were mixed when participants spoke about their experiences of services. Most siblings had at some point been involved either in family therapy sessions or in multi-family therapy. Although most participants found meeting other families or speaking to professionals as helpful, some participants did not feel appropriately involved or considered.

*“[the clinicians] never, like. put my name on the [invitation] letter or anything.” (younger sister)*

*“I don’t think I’ve been involved enough, ‘cause I think especially at the peak of her illness … I think I kept like, everything to myself and I think it would have been better to have somewhere where I could have gone and said what I felt.” (younger sister)*

*“Yeah, I went, I always went to the family therapies, and it was pretty shocking because they didn’t really know what to do with me.” (younger sister)*

### Theme 4: Support for siblings from siblings

Participants expressed unanimously that they felt contact with other siblings with a brother or sister with anorexia nervosa would be the most helpful way to receive support. Siblings described how they felt that only those who had had the same experience could understand them. Some participants expressed that information from specialists and involvement in family therapy helped them understand more and become closer to their families. However, all siblings expressed that what they would find most helpful would be to meet other siblings with similar experiences. Participants described the normalising effect of meeting others in their situation. Also providing a space for them to express how they feel without having to guard what they say for fear of ‘burdening’ others was important for participants.

*“I think having all siblings together you can kind of share what you feel, ‘cause sometimes you just think, ‘Is it normal?’ like, ‘Does it happen to everyone?’ … It’s so, like, extreme that you think maybe it’s just your sibling.” (older sister)*

*“It’s better to talk about it with people in your age group … because you get more of an understanding because you get kind of isolation … it sort of feels like I’m isolated, I’m not part of this because I don’t have a major role in it.” (younger brother)*

## Discussion

This study explored the experiences of brothers and sisters of adolescents with anorexia nervosa, with the aim of furthering understanding of their experiences, their understanding of the illness, and their experiences of service provision. The key findings from this study were that:

- Siblings are negatively and positively affected by a range of emotional, behavioural and relational changes that occurred in the context of their sibling’s eating disorder;
- They often felt unable to talk about their experiences, perceived their needs as secondary, and felt side-lined or misunderstood by services and
- Contact with other siblings was highly valued

These findings are largely consistent with previous findings [9, 16–19, 30, 31] and highlight the need to better support and include siblings in a meaningful way in treatment. The experience can be both challenging and rewarding, with multiple and mixed emotions occurring simultaneously.

Siblings in this study described coping with such pressures by distancing themselves physically and staying out of the family home, seeking ‘normality’ with friends. This is also largely congruent with previous reports [16, 18, 30, 32]. Interventions aimed at families and siblings should pay attention to the effect of siblings internalising such feelings and whether, as expressed in this study, it would be helpful to facilitate a space to share their experiences. This was the most endorsed recommendation by the current sample and may be a useful way of extending current guidance beyond the assessment of siblings’ own needs [23].

Suggestions on how best to include siblings in treatment based on the current findings include (a) a more explicit invitation to treatment and a clearer rationale on when and why to attend, and (b) more direct discussion of the sibling role during recovery and the siblings’ own needs in treatment sessions would help to make sibling involvement feel more useful. Siblings in this study did not say they need, or want, to come to all treatment sessions. Rather for it to be discussed with the whole family and the level of sibling involvement to be based on the needs of each family and their members, not just that of the ill adolescent and/or the parent(s). For some this may include sibling attendance throughout treatment, for others it may be very limited.

Notably, services were experienced as somewhat unresponsive to siblings’ needs, as suggested by parents in previous studies [12, 13]. While some authors have found that siblings are reluctant to attend family therapy [16, 33], our findings suggest such reluctance might be addressed by working with siblings to develop support tailored to their unique needs. One participant noted that even when they attended treatment, the therapist did not seem to know how to include them. This suggests therapists may also need additional guidance on how best to ensure attendance is valued and utilised effectively. Experience of services may also be partly influenced by how well the ill sibling is progressing in treatment. Withers et al. [18] noted that if family treatment was not progressing well, the emotional burden of treatment on siblings was reportedly higher.

There was only one reported difference in the sibling experience based on gender. Sisters, but not brothers, mentioned more self-awareness of their own eating or body image following the development of their sister’s anorexia nervosa. For some, this was associated with feeling watched by their sibling or feeling guilty when eating, for another it helped her appreciate their healthy body shape. While this has been reported previously [16, 31], there is also data indicating that sibling do not struggle with body image more than their peers [34, 35]. This requires future exploration and research.

Although it was not an aim of this study to compare siblings’ experience based on birth order, the ambivalence regarding responsibilities seemed more present for older siblings. Compared to younger siblings, they spoke more explicitly of wanting to support their parents and feeling guilty if they were not able to do so. This is likely influenced by many factors including individual developmental needs and current stage in the family life cycle. Illnesses, including eating disorders, tend to have a centripetal influence, drawing families closer together. While older siblings are likely to be more independent, the current data suggests they are also more strongly pulled into parentified roles of caring for their sibling.

### Limitations

This was a small study and generalisability of the results is limited. Ideally, we would have combined the two pairs of older siblings into one focus group, if it had been clear beforehand that only two people per group would be able to attend—unfortunately this was not the case. The sample included siblings of females with eating disorders only, and the experience of having a brother with an eating disorder remains largely unclear. This sample may have been biased towards those comfortable in groups, and those who had contact with services. Analysis of the data by the same researchers who facilitated the groups may have had the potential to bias the results according to the researchers’ preconceived ideas about what they might find.

Finally, the recruitment strategy was determined by the Research Ethics Committee. Service users who refused consent to contact their siblings did not have to give reasons and, due to data protection rules, it was not possible to analyse differences between those who did and did not give consent. Third party consent in research is a complex issue, but it could be argued that seeking permission from service users before offering siblings a chance to share their experiences undermines siblings’ choices in their own right and reinforces their experience of being marginalised. Further thought should therefore be given to the possibility of contacting siblings directly for future research.

### Conclusion

Data from this study suggest the sibling experience needs to be more carefully considered and included in treatment. Findings point to ways siblings may be better supported, such as peer support groups and a more active involvement in family therapy sessions during which their own needs can be addressed in addition to their siblings’. Specifically, the data indicates an explicit discussion about sibling involvement in treatment is needed, as opposed to a blanket rule of attendance or complete separation of siblings from the treatment process.

## Supplementary Information

**Additional file 1.** Interview topic guide.

## Data Availability

Data are available upon reasonable request.

## Abbreviation

MCCAED: Maudsley Centre for Child and Adolescent Eating Disorders
