# Supplementary material for: Silent witnesses: the experience of having a sibling with anorexia nervosa
Source: J Eat Disord. 2022 Sep 6;10:134. doi: 10.1186/s40337-022-00655-1 (PMC9450355; doi:10.1186/s40337-022-00655-1)
Supplement: Supplementary file 1 — Additional file 1. Interview topic guide. [file 40337_2022_655_MOESM1_ESM.pdf]

Supplementary Material: Topic guide for focus groups

I. How do participants understand their siblings' eating disorders? What do you remember from when you first realised that there was an issue with your sister's eating? How much control do you think your sister had/has over her eating problem? If you had to define eating disorders to another person, what would you say?

II. How does the eating disorder affect family life? What comes to mind when you think of your experience of living with an eating disorder in your family? How have things changed from before the eating disorder to where you are now in your family (e.g. mealtimes, holidays)? What about your parents? How has it affected them? Have things changed between you and your sister? How has it affected your relationship? And other relationships within the family? What have you learnt about your family?

III. How does the eating disorder affect life outside the family? How has the eating disorder affected things in your personal life? Are there any positive things that have resulted from your sister having an eating disorder? Either within or outside of the family?

IV. How can services best support siblings of young people with eating disorders? When your sister started coming here for treatment, were you involved? How about since then? Have you been involved in treatment? What's your opinion of the services your family has come across since the start of your sister's eating disorder? In your experience, what has been helpful/unhelpful? What would you have liked from the services? What suggestions do you have for services? What do you think of siblings' involvement in eating disorder treatment? Is it important for the patient? For the sibling? Why/why not? What advice would you give other siblings' who are going through what you have been through?
